# Optimizing Manufacturing and Osseointegration of Ti6Al4V Implants through Precision Casting and Calcium and Phosphorus Ion Implantation? In Vivo Results of a Large-Scale Animal Trial

**DOI:** 10.3390/ma13071670

**Published:** 2020-04-03

**Authors:** Wölfle-Roos JV, Katmer Amet B, Fiedler J, Michels H, Kappelt G, Ignatius A, Dürselen L, Reichel H, Brenner RE

**Affiliations:** 1 Department of Orthopaedic Surgery, Ulm University, 89081 Ulm, Germany; heiko.reichel@rku.de; 2 Department of Orthopaedic Surgery, Division for Biochemistry of Joint and Connective Tissue Diseases, Ulm University, 89081 Ulm, Germany; betuel.katmer@uni-ulm.de (K.A.B.); joerg.fiedler@uni-ulm.de (F.J.);; 3 Access e.V., 52072 Aachen, Germany; h.michels@access-technology.de; 4 Peter Brehm GmbH, 91085 Weisendorf, Germany; gerhard.kappelt@peter-brehm.de; 5 Institute of Orthopaedic Research and Biomechanics, Ulm University, 89081 Ulm, Germany; anita.ignatius@uni-ulm.de (I.A.); lutz.duerselen@uni-ulm.de (D.L.)

**Keywords:** ion implantation, precision casting, Ti6Al4V, calcium, phosphorus, centrifugal casting

## Abstract

Background: Uncemented implants are still associated with several major challenges, especially with regard to their manufacturing and their osseointegration. In this study, a novel manufacturing technique—an optimized form of precision casting—and a novel surface modification to promote osseointegration—calcium and phosphorus ion implantation into the implant surface—were tested in vivo. Methods: Cylindrical Ti6Al4V implants were inserted bilaterally into the tibia of 110 rats. We compared two generations of cast Ti6Al4V implants (CAST 1st GEN, n = 22, and CAST 2nd GEN, n = 22) as well as cast 2nd GEN Ti6Al4V implants with calcium (CAST + CA, n = 22) and phosphorus (CAST + P, n = 22) ion implantation to standard machined Ti6Al4V implants (control, n = 22). After 4 and 12 weeks, maximal pull-out force and bone-to-implant contact rate (BIC) were measured and compared between all five groups. Results: There was no significant difference between all five groups after 4 weeks or 12 weeks with regard to pull-out force (*p* > 0.05, Kruskal Wallis test). Histomorphometric analysis showed no significant difference of BIC after 4 weeks (*p* > 0.05, Kruskal–Wallis test), whereas there was a trend towards a higher BIC in the CAST + P group (54.8% ± 15.2%), especially compared to the control group (38.6% ± 12.8%) after 12 weeks (*p* = 0.053, Kruskal–Wallis test). Conclusion: In this study, we found no indication of inferiority of Ti6Al4V implants cast with the optimized centrifugal precision casting technique of the second generation compared to standard Ti6Al4V implants. As the employed manufacturing process holds considerable economic potential, mainly due to a significantly decreased material demand per implant by casting near net-shape instead of milling away most of the starting ingot, its application in manufacturing uncemented implants seems promising. However, no significant advantages of calcium or phosphorus ion implantation could be observed in this study. Due to the promising results of ion implantation in previous in vitro and in vivo studies, further in vivo studies with different ion implantation conditions should be considered.

## 1. Introduction

Uncemented implants are widely used in arthroplasty, in primary total hip arthroplasty (THA), up to 80% of implants are uncemented [1]. As the long-term stability of uncemented implants can only be achieved by osseointegration—i.e., a stable connection between the implant surface and the adjacent bone—titanium-based implants are preferred for uncemented implantation due to their high biocompatibility [2,3].

**Figure 1:**
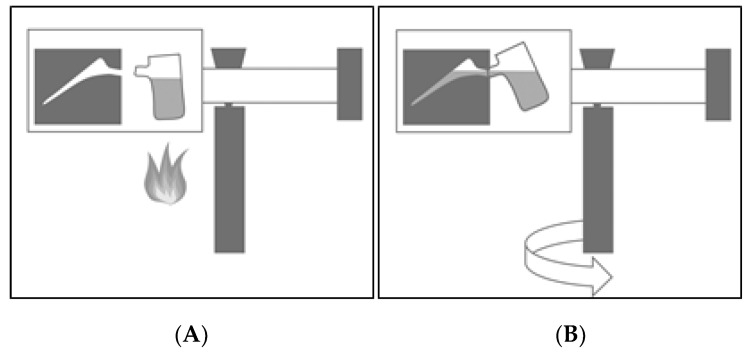
Schematic illustration of a centrifugal precision casting unit: The ingot-casting material is melted (**A**) and the liquid melt is then pressed into the casting mould by rotating the casting arm around a vertical shaft (**B**).

However, the manufacturing process of titanium and its alloys is much more complex than that of cobalt–chromium-based implants [4]. Due to the high melting point of titanium of >1700 °C, its high reactivity to oxygen [5] and its unfavorable fluidity properties [6], precision casting of titanium implants is challenging and currently not routinely used [7]. At present, standard manufacturing of titanium implants involves machining blanks on a milling machine, which results in a high waste of Ti6Al4V material and fabrication costs, as well as a limitation of titanium implants to comparatively simple implant designs [8]. Recent innovations have led to the optimization of centrifugal casting units, see Figure 1. By introducing a cold wall induction crucible and by improving casting conditions, mould material and subsequent heat treatment, centrifugal precision casting has become feasible for the manufacturing of titanium-based implants [9].

**Figure 2:**
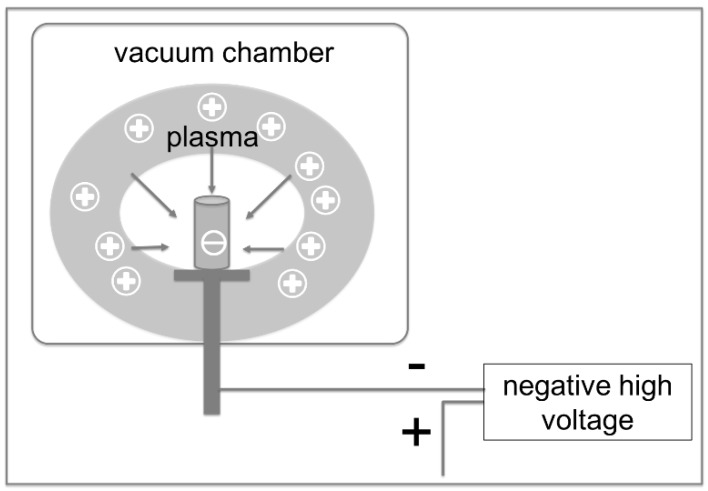
Schematic illustration of plasma immersion ion implantation. In a vacuum chamber, calcium ions are extracted from the plasma—i.e., ionized gas—and accelerated towards the negatively charged implant surface by applying a high voltage direct current.

The technically demanding manufacturing process of titanium-based implants is not the only challenge of uncemented implantation. Osseointegration of the uncemented implant still remains a crucial issue, as the stability of the implantation depends on a strong connection between implant surface and adjacent bone [10]. To enhance osseointegration, countless different methods of surface modification have been developed. One of the most prominent surface modifications is hydroxyapatite coating (HA-coating) [11], which has been widely used in uncemented THA. As, however, delamination of the HA-coating and three-body-wear due to HA-molecules have been increasingly reported in the literature [11,12,13], attempts have been made to implant calcium and phosphorus ions—the chemical components of HA—directly into the implant surface [14], encouraging the formation of calcium phosphate precipitates [15,16]. For complex implant shapes, the so-called plasma immersion ion implantation is currently used [17], see Figure 2.

To improve both the manufacturing and osseointegration of uncemented titanium implants, a novel manufacturing technique—an optimized form of centrifugal precision casting—and a novel surface modification to promote osseointegration—calcium and phosphorus ion implantation into the implant surface—were tested in vivo in this study.

## 2. Methods

### 2.1. Animals

110 adult female WISTAR rats were supplied by Charles River Laboratories (Kißlegg, Germany). They were kept under climate-controlled conditions (21 ± 1.5 °C, 47.5% ± 7.5% humidity, light–dark cycle 12/12 h). Access to food and tap water was ad libitum. Age at the time of surgery was ten weeks; mean body weight was 277 ± 15 g. The animal trial was conducted according to relevant national and international guidelines, such as the ARRIVE guidelines; the study was approved by the Regional Administrative Council (registration number 1246).

### 2.2. Implants

The standard implants consisted of machined, aluminium-oxide-blasted Ti6AlV4 cylindrical rods of 5.2 mm length with a diameter of 1.6 mm (provided by Peter Brehm GmbH, Weisendorf, Germany). One end of the rod was threaded (length 1.2 mm) to enable biomechanical pull-out testing.

All other implants had identical dimensions compared to the standard implants and were manufactured with the optimized centrifugal precision-casting technique, which had been developed in cooperation between the Technical University Aachen (RWTH, Germany), the associated research center Access technology e. V. (Aachen, Germany), and the implant manufacturer Peter Brehm GmbH (Weisendorf, Germany). Implants were cast in a combined vacuum-induction centrifugal casting device Leicomelt 5 TP (ALD Vacuum Technologie GmbH, Hanau, Germany). As the optimized centrifugal precision casting technique is currently undergoing a patent grant procedure, only a general overview of the manufacturing process is given here.

Casting moulds were made of multilayer ceramics with the lost wax technique, with the help of 3D-simulation programs for the casting and solidification process. The ingot-casting material Ti6Al4V was melted in a cold wall induction crucible in a vacuum system with inert gas flushing. The liquid melt was then pressed into the casting mould by rotating the casting arm around a vertical shaft, see Figure 1.

**Figure 3:**
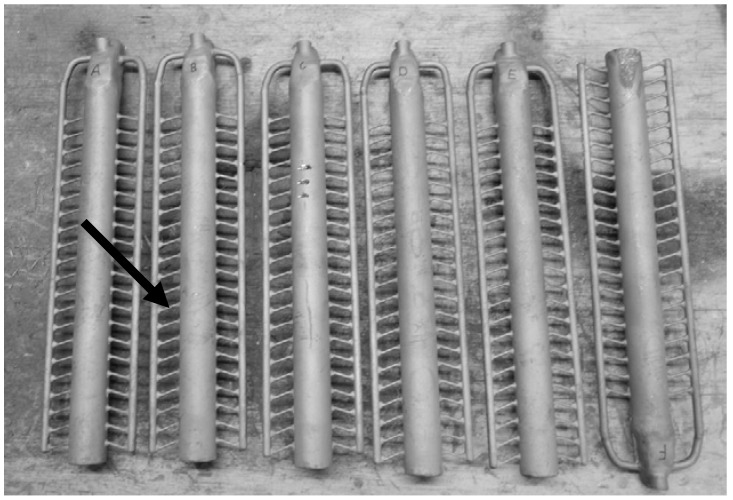
Cast cylindrical implants (→) before separation from the feed channel.

In contrast to other metals’ higher centrifugal forces and higher speed are required for titanium implants to completely fill out the mould due to its low specific weight, high melting point and high solidification speed. After solidification of the melt, the casting was revealed under the destruction of the ceramic form, see Figure 3. The cast implants were separated from the feed channel with a cutting disc. The implants were then submitted to hot isostatic pressing with 920 ± 10 °C at a pressure of 1000 ± 50 bar for 120 ± 30 min to reduce porosity, and subsequent heat treatment.

**Figure 4:**
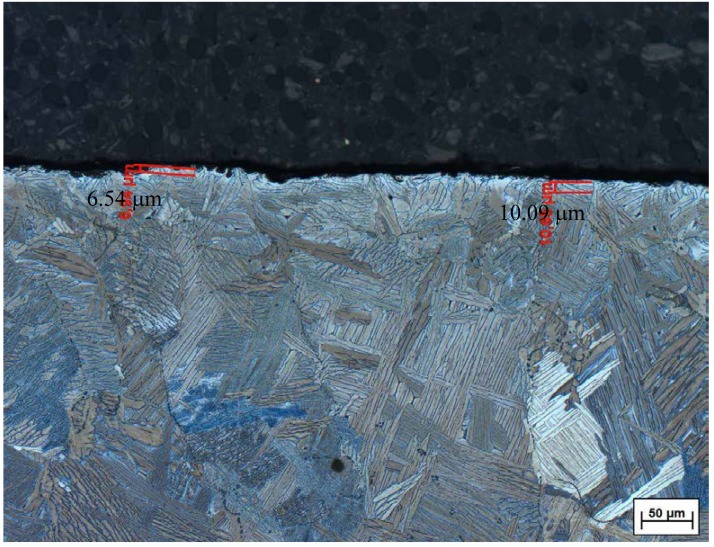
Alpha case layer on the implant surface of cast Ti6Al4V implants of the first generation.

In cast Ti6Al4V implants of the first generation (CAST 1st GEN), the so-called alpha case layer—a hardened brittle layer at the implant surface due to the reaction between the oxides of the mould and the molten titanium, see Figure 4—had to be removed by acid etching at room temperature with Ceramex^®^ (Renfert GmbH, Hilzingen, Germany), i.e., a mixture of 3% hydrofluoric acid and10% sulphuric acid (MediMet Precision Casting and Implants Technology GmbH, Stade, Germany).

By further optimizing the conditions of the casting process, the alpha case layer of cast Ti6Al4V implants of the second generation (CAST 2nd GEN) was negligible, so acid etching could be dispensed with. All cast implants were then submitted to identical aluminium-oxide-blasting to the standard implants. Material analysis showed that the cast material met with the standard grade for Ti6Al4V alloys [4].

Cast Ti6Al4V implants of the second generation were then submitted to plasma immersion ion implantation (PIII) with calcium (CAST + CA group) or phosphorus (CAST + P group) in cooperation with Helmholtz Center Dresden-Rossendorf in a 5-MV-Tandem EGP-10-1 (Efremov-Institut NIIEFA Leningrad, St. Petersburg, Russia). As an ion source, H_3_P, and a calcium arc source, respectively, were used. PIII was conducted at room temperature, ion energy was 30 keV, and ion current 2.4 μA (calcium) and 3–500 μA (phosphorus), respectively, at a dose of 1 × 10^16^/cm^2^.

### 2.3. Experimental Design

The animals were randomly assigned 22 animals each to one of the following groups: (1) control group, (2) CAST 1st GEN, (3) CAST 2nd GEN, (4) CAST + CA, or (5) CAST + P group. According to which group they belonged to, one of the following implants was inserted:

1. Standard Ti6Al4V implants;
2. Cast Ti6Al4V implants of the first generation;
3. Cast Ti6Al4V implants of the second generation;
4. Cast Ti6Al4V implants of the second generation with calcium ion implantation;
5. Cast Ti6Al4V implants of the second generation with phosphorus ion implantation.

Eleven animals of each group were sacrificed after 4 weeks, and the remaining animals 12 weeks after implant insertion. The right tibia was prepared for biomechanical testing and the left tibia for histomorphometric analysis.

### 2.4. Surgical Procedure

The above-mentioned implants were inserted into the proximal tibia on both sides. Anaesthesia was administered by means of an inhalation device (isoflurane 2%) and the subcutaneous injection of analgetics (tramadolor 20 mg/kg) and buprenorphin (0.03 mg/kg). The surgical technique was identical to previous studies conducted in our study group [18]: a 10 mm incision was made at the medial aspect of the proximal tibia, and the periosteum was incised ventrally to the medial collateral ligament. A 1.7 mm drill hole was made level with the insertion of the patella tendon ventrally to the medial collateral ligament using hand-held drills held strictly perpendicular to the longitudinal axis of the tibia. The implant was then inserted into the bone. The threaded part remained outside and was covered by a 2 mm tube cut off from a venous catheter (fluorinated ethylene propylene, Vasofix^®^ Braunüle^®^ 18 G, Braun B., Melsungen, Germany) in order to prevent osseous overgrowth. Postoperative analgesia was ensured by adding tramadolor to the drinking water (25 mg per litre). Antibiotics (clindamycine 45 mg/kg) were administered subcutaneously daily on the first three postoperative days.

### 2.5. Biomechanical Testing

For biomechanical testing, a specifically designed cylindrical device with a matching internal thread was screwed onto the threaded part of the implant of the right tibia. This device was passed through a perforated plate, which served as a mobile fixation of the implant, and then attached to a 200 N load cell (HBM, Darmstadt) of a standard testing machine (Z010, Zwick, Ulm, Germany; see Figure 1A). The surrounding soft tissue around the implant had been purposely left in place to ensure an even contact between the specimen and the perforated plate. The implant was then aligned straight—i.e., longitudinally to the tensile axis—by a low preload of 0.5 N. A force-displacement diagram (test speed 10 mm/s, preload 0.5 N) was recorded by a testing software (testXpert II, Zwick, Ulm, Germany) and the load occurring before the first sudden drop in the tensile force was defined as maximum pull-out force. To calculate the pull-out force per area, a Micro-CT scanning of the former implant bed of the first 44 animals was conducted with a µCT system (Skyscan 1172, Kontich, Belgium). As the correlation between pull-out force and pull-out force per area was found to be highly significant and very strong (Pearson’s correlation coefficient R = 0.970, *p* < 0.001), pull-out force was used as the only biomechanical parameter for the remaining animals.

### 2.6. Histomorphometric Analysis

The left tibia including the inserted implant was embedded in Technovit VLC7200 (Kulzer, Germany) and ground down to sections of 100 μm along the longitudinal axis of the tibia. Masson–Goldner staining of the sections was used to visualize the connective tissue surrounding the implant. The sections were inspected and scanned with a fully automated inverted light microscope (Leica DMI6000B, Wetzlar, Germany). To quantify the amount of bone surrounding the implant, the following parameters were determined semi-automatically with the aid of an imaging analysis software (MetaMorph^®^, Leica, Wetzlar, Germany). The bone-to-implant contact rate (BIC) was calculated by dividing the total length of bone-to-implant contact by the total length around the implant within the tibia. Histomorphometric analysis was conducted by two independent observers blinded to the implant material used.

### 2.7. Statistical Analysis

Statistical advice, including an estimation of required sample size, was gained before the planning of the study based on the only comparable study with a similar animal model and an identical location of the implant insertion [19,20]. For statistical analysis, the Statistical Package for Social Sciences (SPSS^®^ Inc., IBM, version 24) was used. Continuous variables were summarized as mean ± standard deviation. To compare the results of the five groups after 4 and 12 weeks, Kruskal–Wallis test was used. Pearson’s correlation coefficient was used to calculate interobserver reliability and correlation between pull-out force and pull-out force per area. A probability value of less than 0.05 was considered to indicate statistical significance.

## 3. Results

**Figure 5:**
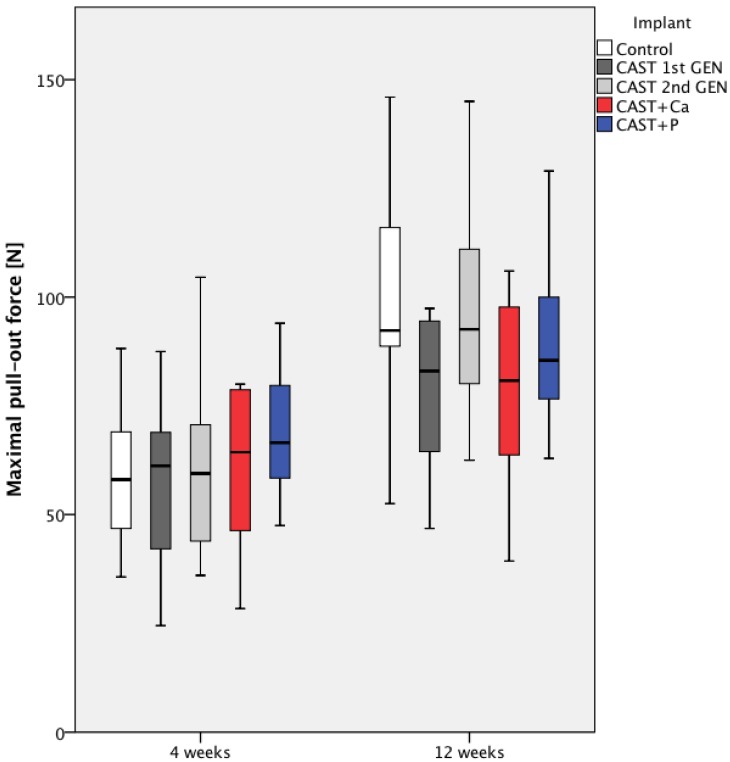
Boxplot of maximum pull-out force after 4 and 12 weeks. The 25th and the 75th percentile as well as the median are indicated by the box, while the whiskers depict maximum and minimum values. No significant difference between all 5 groups was observed (*p* > 0.05, Kruskal–Wallis test).

With regard to maximum pull-out force, there was no significant difference between all five groups after 4 weeks and after 12 weeks (*p* = 0.596 and *p* = 0.127 respectively, Kruskal–Wallis test). After 12 weeks, cast Ti6Al4V implants of the first generation (CAST 1st GEN) showed a slightly lower maximum pull-out force (78.4 ± 16.9 N) compared to the control group (99.8 ± 25.0 N), though this difference did not reach statistical significance. However, no disadvantage of the cast Ti6Al4V implants of the second generation could be observed (95.4 ± 23.0 N) after 12 weeks, see Figure 5.

**Figure 6:**
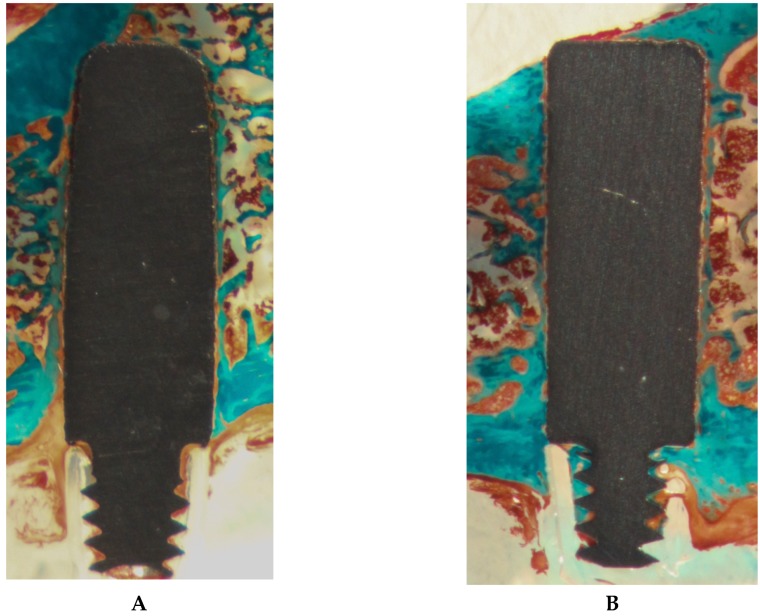
Exemplary Masson–Goldner stains of cast (**A**) and standard (**B**) Ti6l4V implants after 12 weeks. Mature osseous tissue is stained blue, while newly formed bone as well as connective tissue appears red. In both sections, a thin layer of mature bone covers the implant surface.

Analysis of the histological sections showed a thin layer of osseous tissue covering a large part of the implant surface on the section of all five groups. Examples of histologic sections of both cast and machined Ti6Al4V implants are depicted in Figure 6.

**Figure 7:**
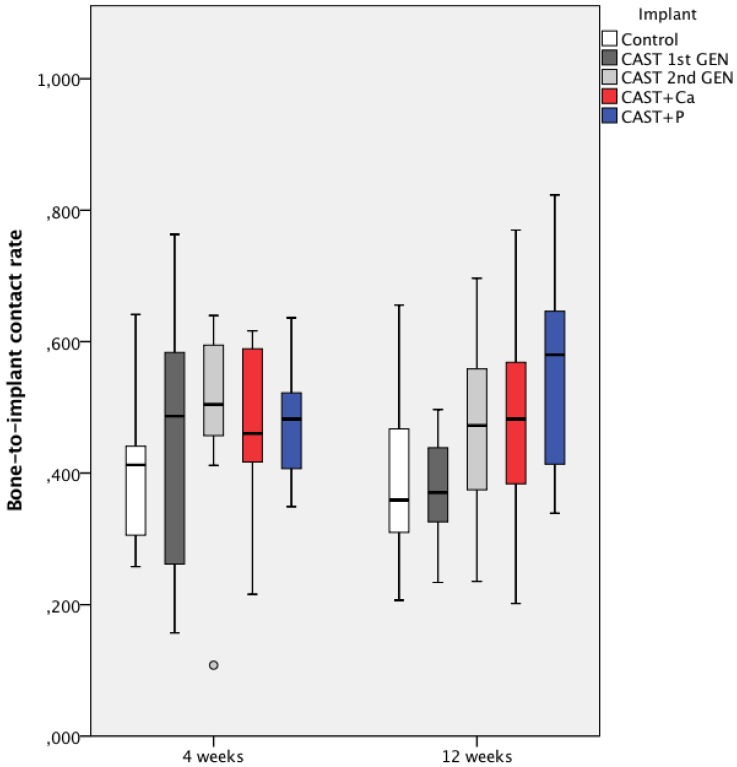
Boxplot of bone-to-implant contact rate (BIC) after 4 and 12 weeks. The 25th and the 75th percentile as well as the median are indicated by the box while the whiskers depict maximum and minimum values. Outlies are plotted as individual dots. There was no significant difference between all five groups after 4 weeks (*p* > 0.05), whereas there was a trend towards higher BIC in the CAST + P group after 12 weeks (*p* = 0.053, Kruskal–Wallis test).

Histomorphometric measurement revealed no significant difference in BIC after 4 weeks between all five groups (*p* > 0.05, Kruskal–Wallis test), whereas there was a trend towards higher BIC in the CAST + P group (54.8% ± 15.2%), especially when compared to the control group (38.6% ± 12.8%) after 12 weeks (*p* = 0.053, Kruskal–Wallis test), see Figure 7.

## 4. Discussion

The most important findings of this study were that no indication of the inferiority of the cast Ti6Al4V implants of the second generation could be observed with regard to osseointegration when compared to standard machined implants, and that no significant advantages after calcium and phosphorus ion implantation could be seen.

### 4.1. Centrifugal Precision Casting

Precision casting of titanium-based implants is technically challenging due to the high melting point of titanium of >1700 °C, its high reactivity to oxygen and its unfavorable fluidity properties. Only in highly specialized centrifugal precision casting units are sufficient centrifugal forces and speed reached to completely fill out the mould, due to the low specific weight and high solidification speed. However, precision-casting of titanium implants holds vast potential economic advantages over the standard milling process [21]. On the one hand, the process offers a significantly decreased material demand per implant by directly casting near net-shape instead of milling away most of the starting ingot, thus greatly lowering the base material demand and cost. Adding to this lower base demand is the inherent improvement in sustainability. Milling waste must be recycled by complex reclaiming processes before it can be reused, while the direct reclamation of casting scrap is regular practice in casting processes, given that constant cast part quality is proven. On the other hand, the centrifugal casting process offers a great freedom of design, in particular by using custom-printed wax patterns in the future, which will enable us to produce implants of complex geometrical shapes (especially those involving undercuts) in large numbers.

Mechanical properties of cast and wrought titanium—the latter being the raw material from which current standard titanium implants are machined—need not necessarily be identical, as the solidification process and the exposure of molten titanium to oxygen varies between the two manufacturing techniques. However, Nastac et al. conducted a review on investment casting of Ti6Al4V alloys and found that almost all the static and dynamic mechanical properties of cast Ti6Al4V alloys are similar to wrought Ti6Al4V. Only the fatigue strength—especially high-cycle fatigue—of cast Ti6Al4V alloys was found to be inferior, which, however, can be improved by subsequent heat treatment [6]. To achieve optimal mechanical properties of cast Ti6Al4V alloys, countless improvements in centrifugal precision casting have been introduced in recent years, e.g., a vacuum system and inert gas flushing due to the high reactivity of titanium with oxygen, high centrifugal forces to ensure complete filling of the mould, or hot isostatic pressing to reduce porosity [6,21]. The alpha case layer—a hardened brittle layer at the implant surface due to the reaction between the oxides of the mould and the molten titanium—still remains a challenge for precision casting of Ti6Al4V [22]. This layer is most commonly removed by acid etching, a technique which in itself bears the risk of hydrogen embrittlement of the implant surface through hydrogen absorption and hydride formation [23]. In our study, we found slightly inferior maximum pull-out force after 12 weeks in cast Ti6Al4V of the first generation, in which the alpha case layer had been removed by acid etching (CAST 1st GEN). By further optimizing the conditions of the casting process, the thickness of the alpha case layer was further reduced (CAST 2nd GEN), so that acid etching could be dispensed with. In these cast Ti6Al4V implants of the second generation, we did not observe any indication of inferiority in comparison to standard Ti6Al4V either on biomechanical testing or on histomorphometric analysis. Standard material testing carried out by our project partners before delivering the implants showed no deviation from standard implant requirements, but further biomechanical studies will have to follow to prove the equivalence of the main mechanical properties such as modulus of elasticity, hardness, toughness, fatigue strength, or tensile strength.

There are only a few in vivo studies comparing cast and machined implants with regard to biocompatibility and osseointegration. Mohammadi et al. implanted cast and machined titanium implants into the abdominal wall of rats so that part of the implant was located in the abdominal wall and part in the peritoneal cavity. On light and electron microscopy they found no difference in peri-implant tissue in the abdominal wall between the two groups. For intraperitoneal implants, significantly more fibroblasts and macrophages were observed on the implant surface of cast implants (in 6/8 implants) when compared to machined implants (in 1/9 implants) [24]. However, as there was a significant difference between tissue response in muscular and peritoneal tissue, it seems doubtful that these findings can predict tissue response in osseous tissue. Moreover, cast titanium implants in their study—as opposed to implants cast with modern centrifugal precision casting units—received hardly any post-processing treatment and showed a comparatively rough implant surface [24]. As this rough implant surface is probably the main reason for the wash-out of titanium particles and the subsequent heightened response of macrophages, their results cannot be compared with modern cast titanium implants. In a more recent study, Mohammadi et al. implanted machined cast titanium implants and standard machined titanium implants into the tibia of rabbits and found no significant difference in bone–implant contact after 3 months (19% BIC in cast implants vs. 25% in machined implants) and 6 months (45% in cast implants vs. 37% in machined implants) between both groups [7]. As in their study cast titanium implants were machined after casting and 0.25 mm of the implant surface were removed, again, their results cannot be compared to ours. There is only one study reporting the application of cast titanium implants in humans: 15 patients received individualized cast titanium implants for the reconstruction of bony skull defects using data from 3D computer tomography scans. There was no control group and mean follow-up was 16.6 months. With the exception of one case of early infection which resulted in the removal of the implant, no complications were noted and osseointegration was successful on clinical and radiological examination [25]. These findings agree with our study, which showed no indication of the inferiority of cast Ti6Al4V implants of the second generation compared to standard Ti6Al4V implants. However, given the small number of patients, the short follow-up, the lack of a control group, and the location of the implants in a non-weight-bearing part of the skeleton [23], evidence regarding the long-term equivalence of cast orthopedic titanium implants when compared to today’s standard implants has yet to be provided. Thus, further in vivo trials involving a large animal model in a biomechanically loaded position might be considered before establishing centrifugal precision casting as a manufacturing technique of titanium orthopedic implants.

### 4.2. Calcium and Phosphorus Ion Implantation

The second part of this study was dedicated to calcium and phosphorus ion implantation into the surface of cast Ti6Al4V implants of the second generation and its effects on osseointegration. Ion implantation represents an ultra-clean process in which the concentration and depth distribution of ions can be controlled with high accuracy [17]. Several in vitro studies have shown the advantageous effects of calcium and phosphorus ion implantation on the mechanical properties of titanium. Corrosion resistance, as measured by electrochemical methods in a simulated body fluid, was significantly increased after calcium and phosphorus ion implantation (ion dose 1 × 10^17^/cm^2^) which is a very important aspect of biocompatibility [26,27]. X-ray photoelectron spectroscopy of calcium and phosphorus implanted titanium (ion dose 1.8 × 10^17^/cm^2^ and 9 × 10^16^/cm^2^ respectively) was conducted to analyze the resulting chemical composition of the implant surface. It was shown that under hydrothermal oxidation a three-step chemical reaction takes place with (1) oxidation of phosphorus ions to P_2_O_5_, (2) hydrolysis resulting in Ca^2+^, PO_4_^3−^ und H^+^, followed by (3) the appearance of needle-like crystallites of calcium phosphates such as hydroxyapatite on the implant surface [16].

The influence of calcium and phosphorus ion implantation on osteogenesis has been thoroughly investigated in vitro by Nayab et al. [28,29,30,31] and Krupa et al. [14,26,27]. Nayab et al. seeded radioisotopically tagged alveolar bone cells on calcium-implanted titanium and found that, although cell adhesion was reduced on the calcium ion-implanted surface, cell spreading and subsequent cell growth of the alveolar bone cells was significantly enhanced [28]. This effect seemed to be dependent on the ion dose: in implants with a high calcium ion dose (1 × 10^17^/cm^2^), the adhesion of MG-63-cells, though initially reduced, was substantially increased in time, and cell spreading was significantly enhanced. In contrast, no marked differences were observed with regard to the adhesion and spreading of MG-63-cells on titanium implanted with low (1 × 10^15^/cm^2^) or medium (1 × 10^16^/cm^2^) ion doses [29]. Gene expression analysis showed the up-regulation of key proteins of osteogenesis such as bone sialoprotein, bone morphogenetic protein receptor-1B and osteopontin in MG-63 cells [30] as well as increased markers of proliferation [31] on calcium-implanted titanium (ion dose 1 × 10^17^/cm^2^). Krupa et al. seeded human mesenchymal stem cells on titanium implanted with calcium ions (ion dose 1 × 10^17^/cm^2^) [26], phosphorus ions (ion dose 1 × 10^17^/cm^2^) [27] and calcium/phosphorus ions combined (ion dose 1 × 10^17^/cm^2^) [14]. In contrast to Nayab et al., they found no positive effects of calcium and/or phosphorus ion implantation on osteogenesis; there was no difference with regard to the vitality of mesenchymal stem cells and the expression of alkaline phosphatase when compared to the control group [14,26,27]. These findings agree with our study, which did not show significant positive effects of either calcium or phosphorus ion implantation on osseointegration.

The effects of ion implantation on osseointegration has only been studied in vivo with calcium-implanted titanium to date. Hanawa et al. investigated bone formation in rats around a titanium implant which had been implanted with calcium ions (ion dose 1 × 10^17^/cm^2^) on one side only; tetracycline and calcein were used as hard-tissue labels. They found more bone formation on the calcium-implanted side of the implant compared to the other side after 2 and 8 days; 18 weeks after surgery, no difference between both sides could be seen [32]. Jinno et al. performed THA in dogs with Ti6Al4V implants with and without calcium ion implantation; doxycycline and fluorescein were used as labels to investigate new bone formation. Histomorphometric analysis showed significantly greater new bone apposition in calcium-implanted stems compared to the control group; 6 and 12 months after implantation no significant difference could be seen between both groups [33]. Cheng et al. inserted cylindrical titanium implants with and without calcium ion implantation into the femur of rats. After 4, 8, and 12 weeks they found significant advantages of calcium-implanted titanium implants on histomorphometric analysis, computer tomographic assessment of the implant bed, and analysis of new bone formation, as well as the biomechanical testing of the push-force when compared to the control group [34]. The findings of these three in vivo studies did not concur with our study, in which no positive effects of calcium ion implantation on osseointegration could be observed. Possibly, the ion density used in our study (1 × 10^16^/cm^2^) was too low, or the ion implantation energy too high (30 keV) to reach enough ion deposition close to the implant surface to allow for any noticeable effects on osseointegration. Furthermore, the properties of the implant surface of the studies mentioned above were not identical when compared to our study, as in some cases polished implants [32] and/or pure titanium implants [32,34] were used. Additionally, the casting process itself may have influenced the metallurgic properties of the implant surface, and thus the depth of ion implantation. As we did not expect a relevant release of implanted ions, serum calcium or phosphorus levels were not determined. This might be included in future studies on the effects of different dosages of ion implantation.

## 5. Conclusions

In this study, we found no indication of inferiority of Ti6Al4V implants cast with the optimized centrifugal precision-casting technique of the second generation compared to standard Ti6Al4V implants. As the employed manufacturing process holds considerable economic potential, mainly due to a significantly decreased material demand per implant by casting near net-shape instead of milling away most of the starting ingot, its application in manufacturing uncemented implants seems promising.

However, no significant advantages of calcium or phosphorus ion implantation could be observed in this study with the doses applied. Due to the promising results of ion implantation in previous in vivo studies and its positive effect on the mechanical properties of titanium implants, further in vivo studies with different ion implantation conditions (regarding ion density and implantation energy) should be considered.
